# Phenotyping Chronic Pain and Neuropathic Pain in Population Studies

**DOI:** 10.1002/ejp.70146

**Published:** 2025-10-08

**Authors:** Ian‐Ju Liang, Dhaneesha N. S. Senaratne, Blair H. Smith

**Affiliations:** ^1^ Chronic Pain Research Group, School of Medicine University of Dundee Dundee Scotland UK

**Keywords:** chronic pain, epidemiology, neuralgia, neuropathic pain, phenotype, prevalence

## Abstract

**Background and Objective:**

Chronic pain is a major global health challenge with substantial individual and societal burden. Epidemiological studies are essential for understanding the scale of the problem and identifying approaches to management. However, they reveal wide variation in reported prevalence, largely due to variations in defining and phenotyping of pain, with estimates spanning roughly 10%–50% depending on wording, recall period, severity thresholds, sampling and ascertainment mode. This paper discusses phenotyping approaches in pain epidemiology, highlighting the need for pre‐specified, transparent case definitions with harmonisation to improve comparability and reproducibility across studies.

**Methods:**

We drew from epidemiological literature on chronic pain phenotyping, focusing on methodological approaches. Examples of successful phenotyping strategies from large cohorts and consortia were identified to illustrate scalable and reproducible methods.

**Results:**

We identified ‘broad and shallow’ versus ‘deep and narrow’ approaches to phenotyping, and how they form part of a pyramid model. We explored this further using neuropathic pain as a worked example, with reference to the International Association for the Study of Pain's Special Interest Group on Neuropathic Pain (NeuPSIG) grading system for neuropathic pain and the Neuropathic Pain Phenotyping by International Consensus (NeuroPPIC) project, which aimed to offer a standardised and scalable phenotype suitable for epidemiological and genetic research. We also briefly reviewed other, modern approaches to phenotyping that have been developed, which combine large population samples, data integration and advanced statistical modelling, with the promise of enhanced comparability, and replication.

**Conclusions:**

Consensus on phenotyping is needed. We have illustrated a structured, scalable approach to standardised phenotyping, supporting data integration, comparability and replication.

**Significance Statement:**

This review highlights the critical importance of standardised phenotyping in chronic pain epidemiology. We introduce the pyramid model as a framework addressing key methodological gaps. Enabling scalable and reproducible phenotyping strengthens the foundation for future research and clinical translation, ultimately improving outcomes for people living with pain.

## 1 Introduction

*One's knowledge of science begins when he can measure what he is speaking about and express it in numbers*.

(Lord Kelvin, 1824–1907, cited in Eysenck (1973)).

Epidemiology, as defined by Last (2001), is *‘*the study of the distribution and determinants of health‐related states or events in specified populations and the application of this study to control health problems’. This definition, though still widely used, has evolved to cover the study of both communicable and non‐communicable conditions across various contexts (Frérot et al. 2018). For individual health conditions, epidemiological methods allow us to explore who is affected, why they occur and how best to reduce the burden of illness. This information is essential for the planning and delivery of healthcare services.

Chronic pain epidemiology, in particular, poses unique challenges due to inconsistencies in phenotyping and case definition. Chronic pain has variably been conceptualised based on physical site affected (e.g., headache), mechanism of pathology (e.g., neuropathic pain), severity (e.g., ‘high impact’ chronic pain) and as a global entity in its own right. This lack of consensus complicates how chronic pain is identified in population studies and contributes significantly to variability in prevalence estimates. The ways in which cases are defined and identified are integral to any epidemiological study.

In this paper, we consider the importance of phenotype and case definitions in studies of chronic pain, and summarise the current state and variability of chronic pain epidemiology across international studies. It is presented as a position piece, aiming to outline key challenges in phenotyping chronic pain, and specifically in neuropathic pain, for population studies, drawing on existing evidence and experience and to propose a conceptual model to support future research. We then focus on phenotyping and its critical role in improving consistency. Using neuropathic pain as an example, we explore the pyramid model for understanding different degrees of phenotyping and discuss how standardised phenotypes can improve replicability when considering different types of research questions. Finally, we briefly consider some newer approaches to phenotyping chronic pain in large‐scale population studies.

## 2 Phenotype, Phenotyping and Case Definition

The word phenotype was coined by the Danish botanist Wilhelm Johannsen (1911), alongside its partner word genotype, to describe ‘the observable properties, characteristics, or form of an organism or person produced by the genotype in synergy or interaction with the environment’ (Johannsen 1911; Porta 2014). However, over the years its use has been adopted beyond genetics into other areas of medicine and science. Thus, in epidemiology the *phenotype* may be considered as the observable set of characteristics of a case. *Phenotyping*, as a verb, is the process of measuring and attributing these characteristics to individual members of the study sample. A *case definition* is ‘a set of criteria…that must be fulfilled in order to identify a person as representing a case of a particular disease or condition’ (Porta 2014), and thus requires the selection of specific components/characteristics from the phenotype to apply in a particular context. Case definitions are often specific to individual studies and form the basis of case identification.

## 3 Case Definition Matters

Estimates of chronic pain prevalence vary widely across studies, populations and countries. A recent systematic review by Rometsch et al. (2025), covering over 860,000 European adults, reported wide‐ranging estimates: point prevalence from 12% to 48%, 6‐month prevalence from 17.5% to 49.8%, 12‐month prevalence from 8.1% to 44.6% and lifetime prevalence from 12.7% to 33.7%. The authors concluded that such variability could be reduced by applying consistent diagnostic criteria and standardised assessment tools, such as structured interviews and validated pain scales. Beyond European populations, global estimates show similar variability. Data from the World Health Survey, covering 52 countries and five continents, demonstrated an overall pain prevalence of 27.5%, with a range across countries from 9.9% to 50.3% (lowest in China, highest in Morocco) (Zimmer et al. 2022). Another systematic review identified prevalence studies from 34 countries and found an overall chronic pain prevalence of approximately 30.3%, with slightly higher prevalence in countries that have a lower Human Development Index (HDI) (33.9%; range 5.5% in Nigeria to 60.4% in Ukraine) compared with higher‐HDI countries (29.9%; range 8.7% in Singapore to 53.7 in Sweden) (Elzahaf et al. 2012). Using the World Bank regional classification, the authors also found that prevalence was highest in North America (35.6%) and sub‐Saharan Africa (40.8%), moderate in Europe/Central Asia (29.2%) and the Middle East/North Africa (28.0%) and lower in East/South Asia (19%–25%) and Latin America/Caribbean (26.4%). Similarly, a systematic review focusing on low‐ and middle‐income countries reported a pooled prevalence of unspecified chronic pain of 34%, with a range of 13%–49% and the authors attributed much of this variation to unstandardised case definition and phenotyping (Jackson et al. 2016). These studies highlight that differences in how chronic pain is defined, measured and categorised account for much of the observed variability across populations.

Population prevalence estimates are highly sensitive to variations in case definition and the methods used to collect phenotype data. For example:

- James et al. (1991) performed an interview‐based study with 1498 participants from urban populations in New Zealand and reported that the lifetime prevalence of life‐disrupting pain was over 80%.
- Elliott et al. (1999) used a broad case definition (any pain or discomfort present for at least 3 months) but also included pain location and severity grading (Von Korff et al. 1992) to allow further categorisation. They estimated a population prevalence of any chronic pain of 46.5%, with 6.3% reporting the most severe grade (Smith et al. 2001). Chronic pain and severe chronic pain were associated with higher age and greater deprivation, among other factors.
- Breivik et al. (2006) conducted a large‐scale European telephone interview survey using a definition of pain lasting 6 months or more and rated ≥ 5/10 in intensity, experienced in the most recent month, occurring at least twice in the most recent week. They estimated that 19% of adults across 15 European countries and Israel (ranging from 12% in Spain to 30% in Norway) were living with chronic pain based on their screening criteria.
- The UK Biobank research cohort (Sudlow et al. 2015) used a baseline questionnaire that assessed pain lasting more than 3 months in seven body sites, or ‘all over the body’, which interfered with usual activities. With this, McQueenie et al. (2021) found a prevalence of pain in at least one site of 43.7%, and of pain in more than three sites of 4.4%. They found a strong association between chronic pain and multimorbidity, indicating that individuals with more than four long‐term conditions were over 20 times more likely to report widespread pain than those with none.
- Baskozos et al. (2023) applied an updated re‐phenotyping approach in the UK Biobank, using participant responses aligned to the International Classification of Diseases 11th Revision (ICD‐11) (Treede et al. 2019) along with the *Douleur Neuropathique en 4 Questions* (DN4) questionnaire (Bouhassira et al. 2005) to classify neuropathic versus non‐neuropathic chronic pain. Among 148,828 participants, 51.1% reported chronic pain, with 9.2% classified as neuropathic. The study showed that neuropathic pain was associated with poorer health, higher pain intensity, and comorbid conditions such as diabetes, fibromyalgia and musculoskeletal disorders.
- Rikard et al. (2023) used a subsection of the National Health Interview Survey in the United States that sought to define chronic pain as pain on ‘most days or every day’ (rather than ‘never or some days’) during the previous 3 months. They extrapolated from an interviewed sample of 29,482 adults to estimate a national prevalence of 20.9% in 2021, and 6.9% experiencing high‐impact chronic pain (limiting daily activities or work on most or all days).

However, even with harmonised definitions, other aspects of study design, including sampling and recruitment methods, can influence prevalence estimates. For example, some high estimates may reflect specific urban samples or interview‐based methodologies; others may be affected by non‐population‐based sampling, self‐report questionnaires, or volunteer bias in research cohorts. Although these studies provide insights and increasingly robust phenotyping, their generalisability may be limited by such methodological factors. Furthermore, some research cohorts (e.g., UK Biobank) display a “healthy volunteer” selection bias and often under‐represent priority populations who disproportionately bear the burden of chronic pain (Fry et al. 2017). These selection biases may therefore limit the broader applicability of findings. In addition, because most biobank studies are conducted in high‐income settings, their results may not fully translate to middle‐ and low‐income contexts (Brayne and Moffitt 2022). For example, in the study reported by Zimmer et al. (2022), the same chronic pain definition was used in all 52 countries, yet prevalence still ranged widely. This variation was explained by a model incorporating six factors (i.e., equality, population density, gender inequality, life expectancy and global region), emphasising that determinants beyond phenotyping also influence prevalence estimates.

Regardless, the above examples highlight how even subtle differences in wording, recall periods, or severity thresholds can lead to substantially different population estimates. Does this matter? The lack of standardisation in case definitions has real‐world consequences. Inconsistent or unreliable data complicates public health planning, hampers the development of targeted treatments, and limits our ability to evaluate the effectiveness of healthcare interventions and systems. Furthermore, these challenges extend beyond simple prevalence estimates. Evaluating the burden of disease also requires consistency in case definition. Chronic pain is a multifactorial condition that seldom occurs in isolation, frequently coexisting with other long‐term conditions. It significantly affects psychological, emotional and social wellbeing; individuals with chronic pain are more likely to experience mood disorders, including depression, anxiety and suicide (Aaron et al. 2025; McQueenie et al. 2021). Similarly, studies have shown that chronic pain is associated with a higher burden of comorbidities that interact in complex ways to worsen outcomes and contribute to a cycle of disability and distress (Dominick et al. 2012; Dueñas et al. 2016). Among older adults, pain‐related disability often leads to social isolation and diminished community engagement (Currie and Wang 2004). Van Hecke et al. (2017) also demonstrated the shared genetic predispositions underlying chronic pain and common comorbidities. Without a unified framework, we risk both under‐ and over‐estimating the true burden of chronic pain. Moreover, this variability undermines efforts to identify consistent biological, psychological and social correlates of pain, constraining the development of tailored interventions.

## 4 Improving Consistency

Given the importance of consistent definitions, one significant step toward standardisation has come from the reclassification of chronic pain in the ICD‐11, a collaborative process between the World Health Organization (WHO) and the International Association for the Study of Pain (IASP). ICD‐11 defines chronic pain as pain persisting or recurring for more than 3 months and distinguishes between chronic primary pain (e.g., fibromyalgia) and chronic secondary pain (e.g., chronic cancer‐related pain or chronic neuropathic pain) (Treede et al. 2019). This internationally agreed framework represents a valuable foundation for clinical and research use. The hierarchy will take some time to bed into existing clinical and research processes, but it will provide a useful structure for consistency in diagnostic coding, epidemiological tracking and health policy. However, ICD‐11 was primarily designed as a clinical tool, relying on clinician expertise and one‐to‐one assessment. The challenge lies in adapting and expanding these definitions for population research, where consistent, replicable approaches to diagnosis and phenotyping are required at scale.

Protocols adopted by large research cohorts may provide another means toward consistency. Consortia such as DOLORisk (Pascal et al. 2019), PAINSTORM (n.d.) and efforts within UK Biobank have adopted consistent data collection and phenotyping frameworks. These initiatives may reduce heterogeneity by implementing unified strategies to classify chronic pain, particularly subtypes such as neuropathic pain (Hébert et al. 2023).

## 5 Approaches to Phenotyping and the Pyramid Model

At the core of this variability lies a deceptively simple question: what do we mean by “chronic pain”? It is only relatively recently that we have settled on the time‐based definition of pain lasting longer than 3 months (Treede et al. 2019). Prior to this consensus agreement, operationalising the concept of chronic pain was not straightforward, and the way it was identified profoundly influenced study outcomes.

Phenotyping study participants can be considered as a spectrum, ranging from ‘broad and shallow’ (i.e., collecting small amounts of data from a large number of participants) to ‘narrow and deep’ (i.e., collecting large amounts of data from a small number of participants). This reflects approaches to phenotyping that have been widely used and discussed previously in epidemiology (Robinson 2012; Swerdel and Conover 2023). There are a large number of variables that could potentially be collected; phenotypic characteristics include pain duration, characteristics, intensity, location, associated symptoms, interference with function, underlying mechanisms or pathology, examination findings, investigation results, response to treatment and so on. These variables may be obtained through different processes; for example, through participant interviews/surveys, clinical examination, clinical investigations, or evaluation of medical records. Often the decision about what type of phenotyping to perform for a given study is a pragmatic compromise driven by limitations in key resources (i.e., time, money, or people) or by study design. More detailed phenotypes may be more scientifically rigorous but are difficult to implement at scale, whilst simple phenotypes are easier to collect but may lack precision. This trade‐off between validity and feasibility is a common dilemma in population‐based research.

**FIGURE 1:**
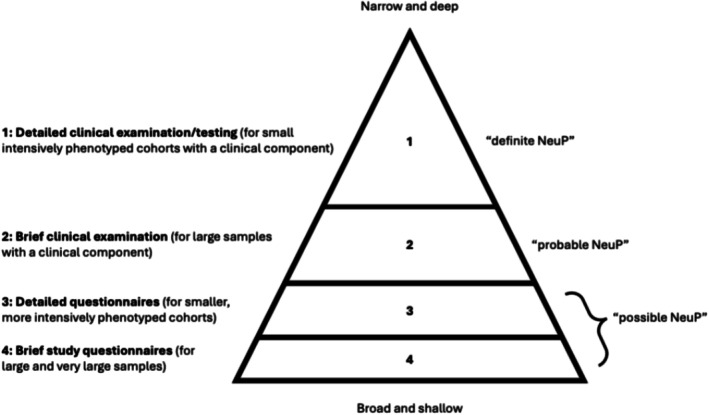
Phenotyping chronic (neuropathic) pain for human studies. NeuP, neuropathic pain.

This concept forms the basis for the pyramid model of phenotyping (Figure 1). The base of the pyramid reflects the ‘broad and shallow’ approach: using brief questionnaires across large or very large study populations. At each higher level, the amount of data collected increases whilst the sample size falls, until the uppermost level (‘narrow and deep’) where detailed clinical examination and/or testing can only be done on small study populations.

Although the frameworks we discuss may apply broadly to chronic pain, we will focus on neuropathic pain as a worked example to illustrate the principles of phenotyping and the pyramid model.

## 6 Using the Pyramid Model to Explore Neuropathic Pain

The issue of phenotyping is particularly challenging for neuropathic pain, which is variably classified in the literature. Clinical features such as sensory loss or positive signs (e.g., allodynia, hyperalgesia) are difficult to measure and rarely available. Overly simplified phenotypes may obscure biologically meaningful pain subtypes, hindering research progress and clinical translation. Without a common case definition, meaningful comparisons or data pooling across studies remain difficult. For example, a systematic review of genetic studies in neuropathic pain (Veluchamy et al. 2018) identified 29 different case definitions across 29 studies, resulting in minimal overlap of findings and poor replication of genetic associations. Importantly, even within the same dataset, small changes in case definition led to markedly different genetic associations. In a genome‐wide association study (GWAS), Meng, Deshmukh, Donnelly, et al. (2015) identified neuropathic pain cases according to recorded prescriptions of anti‐neuropathic pain medicines, and reported a possible sex‐specific genetic variant on chromosome 1. In the same dataset, the addition of recorded monofilament testing of foot sensation to the case definition did not find this suggestive association but found a different one (not sex‐specific) on chromosome 8 (Meng, Deshmukh, Donnelly, et al. 2015). This highlights the sensitivity of genetic discovery to phenotyping approaches and the need for uniformity. The solution lies in developing and adopting phenotypes that strike the right balance: valid enough to capture relevant clinical and biological features, yet feasible to apply in large‐scale datasets. Although our worked example focuses on neuropathic pain, the principles described below may be applicable to other types of chronic pain which may have other (possibly greater) biological or genetic complexities.

Recent initiatives have begun addressing this gap, aiming to standardise neuropathic pain phenotyping across clinical and research cohorts, in order to improve comparability, reproducibility and clinical relevance. The IASP's Special Interest Group on Neuropathic Pain (NeuPSIG) developed a grading system for neuropathic pain (Finnerup et al. 2016) that parallels the pyramid model of phenotyping (Figure 1). In the clinical context, evaluation begins with a simple clinical history aiming to discern the presence of a pain distribution that is consistent with a lesion or disease of the somatosensory system, leading to an assessment of ‘possible neuropathic pain’. In the research context, this could also be done with detailed or simple questionnaires, potentially involving large or very large numbers of people (equivalent to levels three and four of the pyramid model). The addition of examination findings (sensory signs in the same neuroanatomical distribution) leads to an assessment of ‘probable neuropathic pain’. In the clinical context, the examination naturally follows from the history, but in the research context, adding these additional data requires a substantially different study design (with a corresponding increase in resource requirements) and so will likely be associated with smaller sample sizes (level two of the pyramid model). Finally, confirmatory tests (such as neurophysiology or imaging) that demonstrate a lesion or disease provide evidence for ‘definite neuropathic pain’, and in the context of research, will require yet another step up in resources (corresponding to level one of the pyramid model).

## 7 Neuropathic Pain Phenotyping by International Consensus (NeuroPPIC)

**FIGURE 2:**
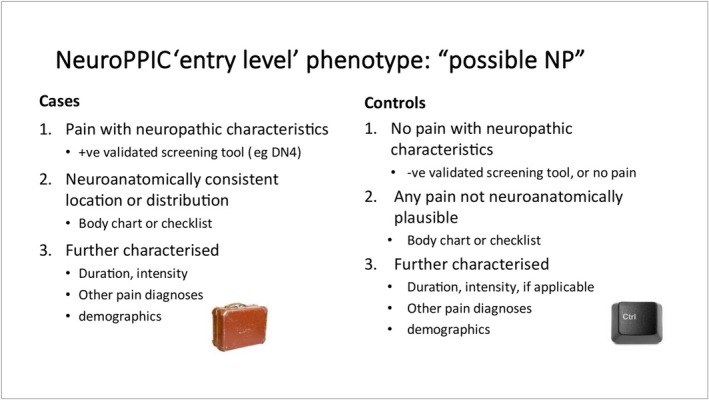
NeuroPPIC ‘entry level phenotype’: ‘possible neuropathic pain’.

NeuPSIG undertook the Neuropathic Pain Phenotyping by International Consensus (NeuroPPIC) project, which aimed to offer a standardised and scalable phenotype suitable for epidemiological and genetic research. The main output was a consensus‐driven, pragmatic ‘entry‐level’ phenotype to enable harmonisation across large‐scale datasets (Van Hecke et al. 2015). Its core components were chronicity (pain > 3 months), neuroanatomic distribution of pain, validated screening tool results (e.g., DN4 (Bouhassira et al. 2005)), assessing pain characteristics and the introduction of severity ratings as required. The ‘entry‐level’ phenotype could be applied in population cohorts without requiring clinical examination or advanced diagnostics (Figure 2).

This is intended to form a basis for a hierarchical approach to phenotyping, mirroring the NeuPSIG grading system (Finnerup et al. 2016), with diagnostic certainty becoming possible as additional examination and tests are applied. ‘Possible neuropathic pain’ is based on symptoms and screening tools. Further phenotyping will allow progression toward ‘probable neuropathic pain’, with examination confirming sensory signs in the same neuroanatomically plausible distribution and ‘definite neuropathic pain’ with the addition of confirmatory tests. This phenotyping should also align with the more recent classification of neuropathic pain in ICD‐11 (Scholz et al. 2019), including peripheral (e.g., polyneuropathy, radiculopathy) and central (e.g., spinal cord injury, multiple sclerosis) neuropathic pain.

NeuroPPIC's application in cohorts such as Genetics of Diabetes Audit and Research in Tayside Scotland (GoDARTS, (Hébert et al. 2018)) and Generation Scotland: Scottish Family Health Study (GS:SFHS, (Smith et al. 2013)) exemplifies this level, where pain status was inferred using brief questionnaire instruments, as part of the DOLORisk programme (Hébert et al. 2021; Pascal et al. 2019). A GWAS meta‐analysis of the GoDARTS and GS:SFHS datasets (1244 cases and 2832 controls) identified genome‐wide significant associations with variants in the ephrin receptor tyrosine kinase A3 (*EPHA3*) gene, which is implicated in nervous system development and synaptic plasticity. Narrow‐sense heritability of neuropathic pain was estimated at 33%, further supporting a substantial genetic contribution to neuropathic pain and reinforcing the utility of the NeuroPPIC phenotype (Veluchamy et al. 2021).

Moving up the pyramid, the DOLORisk consortium and collaborators utilised this approach by adding measures of pain intensity and findings of detailed clinical examination to the ‘entry level’ NeuroPPIC phenotype in a harmonised cohort of people with diabetic neuropathy (Hébert et al. 2021; Pascal et al. 2019). This allowed the identification, in a GWAS, of a potassium channel gene (*KCNT2*) associated with ‘probable neuropathic pain’ in diabetic neuropathy (Åkerlund et al. 2025). Further robust phenotyping, using quantitative sensory testing, allowed a case definition approaching ‘definite neuropathic pain’ and representing the peak of the pyramid (Figure 1). This step showed that the identified *KCNT2* variant was associated with significantly different responses to testing of mechanical sensitivity. This study illustrates the value of a harmonised and stratified approach to phenotyping, with valuable insights gained from each stage of the pyramid.

## 8 Other Phenotyping Approaches for Chronic Pain

We have focused on questionnaire‐based phenotyping approaches, particularly for large population studies, at the base of the proposed pyramid. Other approaches to phenotyping neuropathic pain in large samples include coded diagnoses in clinical datasets (e.g., Gajria et al. 2011; Hall et al. 2006), and the use of prescribing records (McDermott et al. 2006; Veluchamy et al. 2021).

When pain phenotypes cannot be directly harmonised, other approaches are possible. For example, Meng et al. (2023) reported a GWAS meta‐analysis on the self‐reported headache phenotype from UK Biobank, and the self‐reported migraine phenotype from 23andMe (a genomics company with a large research participant database). This was considered a valid approach despite the differences between these phenotypes, as they identified a strong genetic correlation before the meta‐analysis was performed (*r* = 0.72 (*p* = 1.66 × 10–68, standard error = 0.04)). This approach allowed identification of four new genetic loci associated with headache, in addition to the previously identified 34 loci from individual studies included. In the study by Veluchamy et al. (2021) described above, additional cases of neuropathic pain (*n* = 3268) were identified through self‐reported prescribing. These were meta‐analysed with those identified in GoDARTS and GS:SFHS in a further GWAS, with the identification of another biologically plausible genetic association, of genome‐wide significance, on chromosome 12. Their comparison of questionnaire‐based and prescribing‐based phenotypes confirmed reasonable sensitivity and specificity.

Beyond conventional approaches, increasingly sophisticated phenotyping approaches incorporate complex genetic and biopsychosocial factors that transcend the pyramid's restrictions. For instance, Zorina‐Lichtenwalter et al. (2023) integrated questionnaire‐based data with existing medical records and applied genomic structural equation modelling to 24 chronic pain conditions (from a list of 91 candidate phenotypes) in the UK Biobank, identifying shared genetic patterns across conditions, driven by two latent factors (i.e., one general and another specific to musculoskeletal pain). Similarly, Tanguay‐Sabourin et al. (2023) combined data from questionnaires, laboratory, imaging and genomic datasets to develop a multidimensional biopsychosocial framework for characterising chronic pain. These studies illustrate how harmonisation and multimodal data integration can develop complex phenotypes, reveal common mechanisms and shared features across chronic pain conditions. These multidimensional, scalable frameworks can enhance prediction and understanding of widespread and high‐impact pain. Together, these recent studies highlight the value of integrating broad‐scale genomic insights with detailed phenotyping and biopsychosocial models, indicating the need for flexible, layered frameworks to capture the multifaceted nature of chronic pain. They demonstrate that modern approaches to phenotyping allow detailed examination even in very large cohorts.

## 9 Conclusions

Epidemiological research has revealed the enormous burden of chronic pain, but has also exposed critical methodological limitations, particularly the inconsistency in how pain is defined and identified. Prevalence estimates vary widely across studies, often due to differences in case definitions, phenotyping approaches and population characteristics.

Phenotyping is central to pain epidemiology, yet it is frequently underappreciated and inconsistently applied. In response to calls for harmonisation of phenotyping approaches, we have described some approaches and given examples of their success. These approaches support scalability, reproducibility, meta‐analysis and cross‐cohort comparability, enabling researchers to select phenotyping strategies suited to their study design and resources.

As data linkage, machine learning and digital health tools advance, future pain research will increasingly rely on such standardised and scalable phenotyping approaches. To maximise the utility of epidemiological data and support progress toward personalised pain medicine, the field must move toward consensus. Standardised phenotyping, anchored in initiatives like NeuroPPIC and informed by frameworks such as the pyramid model, will enable replication, accelerate discovery and ultimately improve outcomes for people living with chronic pain.

## Author Contributions

The paper was conceived by BHS and jointly written by all authors. All authors discussed the results and commented on the manuscript.

## Conflicts of Interest

The authors declare no conflicts of interest.

## References

[ejp70146-bib-0001] Aaron, R. V. , S. G. Ravyts , N. D. Carnahan , et al. 2025. “Prevalence of Depression and Anxiety Among Adults With Chronic Pain: A Systematic Review and Meta‐Analysis.” JAMA Network Open 8, no. 3: e250268. 40053352 10.1001/jamanetworkopen.2025.0268 PMC11889470

[ejp70146-bib-0002] Åkerlund, M. , G. Baskozos , W. Li , et al. 2025. “Genetic Associations of Neuropathic Pain and Sensory Profile in a Deeply Phenotyped Neuropathy Cohort.” Pain 166, no. 6: 1354–1368. 39471050 10.1097/j.pain.0000000000003463 PMC12067614

[ejp70146-bib-0003] Baskozos, G. , H. L. Hébert , M. M. Pascal , et al. 2023. “Epidemiology of Neuropathic Pain: An Analysis of Prevalence and Associated Factors in UK Biobank.” Pain Reports 8, no. 2: e1066. 37090682 10.1097/PR9.0000000000001066 PMC7614463

[ejp70146-bib-0004] Bouhassira, D. , N. Attal , H. Alchaar , et al. 2005. “Comparison of Pain Syndromes Associated With Nervous or Somatic Lesions and Development of a New Neuropathic Pain Diagnostic Questionnaire (DN4).” Pain 114, no. 1–2: 29–36. 15733628 10.1016/j.pain.2004.12.010

[ejp70146-bib-0005] Brayne, C. , and T. E. Moffitt . 2022. “The Limitations of Large‐Scale Volunteer Databases to Address Inequalities and Global Challenges in Health and Aging.” Nature Aging 2, no. 9: 775–783. 37118500 10.1038/s43587-022-00277-x PMC10154032

[ejp70146-bib-0006] Breivik, H. , B. Collett , V. Ventafridda , R. Cohen , and D. Gallacher . 2006. “Survey of Chronic Pain in Europe: Prevalence, Impact on Daily Life, and Treatment.” European Journal of Pain 10, no. 4: 287–333. 16095934 10.1016/j.ejpain.2005.06.009

[ejp70146-bib-0007] Currie, S. R. , and J. Wang . 2004. “Chronic Back Pain and Major Depression in the General Canadian Population.” Pain 107, no. 1–2: 54–60. 14715389 10.1016/j.pain.2003.09.015

[ejp70146-bib-0008] Dominick, C. H. , F. M. Blyth , and M. K. Nicholas . 2012. “Unpacking the Burden: Understanding the Relationships Between Chronic Pain and Comorbidity in the General Population.” Pain 153, no. 2: 293–304. 22071318 10.1016/j.pain.2011.09.018

[ejp70146-bib-0009] Dueñas, M. , B. Ojeda , A. Salazar , J. A. Mico , and I. Failde . 2016. “A Review of Chronic Pain Impact on Patients, Their Social Environment and the Health Care System.” Journal of Pain Research 28, no. 9: 457–467. 10.2147/JPR.S105892 PMC4935027 27418853

[ejp70146-bib-0010] Elliott, A. M. , B. H. Smith , K. I. Penny , W. C. Smith , and W. A. Chambers . 1999. “The Epidemiology of Chronic Pain in the Community.” Lancet 354, no. 9186: 1248–1252. 10520633 10.1016/s0140-6736(99)03057-3

[ejp70146-bib-0011] Elzahaf, R. A. , O. A. Tashani , B. A. Unsworth , and M. I. Johnson . 2012. “The Prevalence of Chronic Pain With an Analysis of Countries With a Human Development Index Less Than 0.9: A Systematic Review Without Meta‐Analysis.” Current Medical Research and Opinion 28, no. 7: 1221–1229. 22697274 10.1185/03007995.2012.703132

[ejp70146-bib-0012] Eysenck, H. J. 1973. The Measurement of Intelligence. Medical and Technical Publishing Co.

[ejp70146-bib-0013] Finnerup, N. B. , S. Haroutounian , P. Kamerman , et al. 2016. “Neuropathic Pain: An Updated Grading System for Research and Clinical Practice.” Pain 157, no. 8: 1599–1606. 27115670 10.1097/j.pain.0000000000000492 PMC4949003

[ejp70146-bib-0014] Frérot, M. , A. Lefebvre , S. Aho , P. Callier , K. Astruc , and L. S. Aho Glélé . 2018. “What Is Epidemiology? Changing Definitions of Epidemiology 1978‐2017.” PLoS One 13, no. 12: e0208442. 30532230 10.1371/journal.pone.0208442 PMC6287859

[ejp70146-bib-0015] Fry, A. , T. J. Littlejohns , C. Sudlow , et al. 2017. “Comparison of Sociodemographic and Health‐Related Characteristics of UK Biobank Participants With Those of the General Population.” American Journal of Epidemiology 186, no. 9: 1026–1034. 28641372 10.1093/aje/kwx246 PMC5860371

[ejp70146-bib-0016] Gajria, C. , J. Murray , R. Birger , et al. 2011. “Identification of Patients With Neuropathic Pain Using Electronic Primary Care Records.” Journal of Innovation in Health Informatics 19, no. 2: 83–90. 10.14236/jhi.v19i2.799 22417818

[ejp70146-bib-0017] Hall, G. C. , D. Carroll , D. Parry , and H. J. McQuay . 2006. “Epidemiology and Treatment of Neuropathic Pain: The UK Primary Care Perspective.” Pain 122, no. 1: 156–162. 16545908 10.1016/j.pain.2006.01.030

[ejp70146-bib-0018] Hébert, H. L. , M. M. Pascal , B. H. Smith , D. Wynick , and D. L. Bennett . 2023. “Big Data, Big Consortia, and Pain: UK Biobank, PAINSTORM, and DOLORisk.” Pain Reports 8, no. 5: e1086. 38225956 10.1097/PR9.0000000000001086 PMC10789453

[ejp70146-bib-0019] Hébert, H. L. , B. Shepherd , K. Milburn , et al. 2018. “Cohort Profile: Genetics of Diabetes Audit and Research in Tayside Scotland (GoDARTS).” International Journal of Epidemiology 47, no. 2: 380–381j. 29025058 10.1093/ije/dyx140 PMC5913637

[ejp70146-bib-0020] Hébert, H. L. , A. Veluchamy , G. Baskozos , et al. 2021. “Cohort Profile: DOLORisk Dundee: A Longitudinal Study of Chronic Neuropathic Pain.” BMJ Open 11, no. 5: e042887. 10.1136/bmjopen-2020-042887 PMC8103377 33952538

[ejp70146-bib-0021] Jackson, T. , S. Thomas , V. Stabile , X. Han , M. Shotwell , and K. McQueen . 2016. “Prevalence of Chronic Pain in Low‐Income and Middle‐Income Countries: A Systematic Review and Meta‐Analysis.” Anesthesia and Analgesia 123, no. 3: 739–748. 27537761 10.1213/ANE.0000000000001389

[ejp70146-bib-0022] James, F. R. , R. G. Large , J. A. Bushnell , and E. J. Wells . 1991. “Epidemiology of Pain in New Zealand.” Pain 44, no. 3: 279–283. 2052397 10.1016/0304-3959(91)90097-H

[ejp70146-bib-0023] Johannsen, W. 1911. “The Genotype Conception of Heredity.” American Naturalist 45, no. 531: 129–159.

[ejp70146-bib-0024] Last, J. M. 2001. Dictionary of Epidemiology. 4th ed. Oxford University Press.

[ejp70146-bib-0025] McDermott, M. E. , B. H. Smith , A. M. Elliott , C. M. Bond , P. C. Hannaford , and W. A. Chambers . 2006. “The Use of Medication for Chronic Pain in Primary Care, and the Potential for Intervention by a Practice‐Based Pharmacist.” Family Practice 23, no. 1: 46–52. 16107494 10.1093/fampra/cmi068

[ejp70146-bib-0026] McQueenie, R. , B. D. Jani , S. Siebert , et al. 2021. “Prevalence of Chronic Pain in LTCs and Multimorbidity: A Cross‐Sectional Study Using UK Biobank.” Journal of Multimorbidity and Comorbidity 11: 26335565211005870. 35004337 10.1177/26335565211005870 PMC8728767

[ejp70146-bib-0027] Meng, W. , H. Deshmukh , N. Van Zuydam , et al. 2015. “A Genome‐Wide Association Study Suggests an Association of C hr8p21.3 (GFRA2) With Diabetic Neuropathic Pain.” European Journal of Pain 19, no. 3: 392–399. 24974787 10.1002/ejp.560 PMC4737240

[ejp70146-bib-0028] Meng, W. , H. A. Deshmukh , L. A. Donnelly , et al. 2015. “A Genome‐Wide Association Study Provides Evidence of Sex‐Specific Involvement of Chr1p35. 1 (ZSCAN20‐TLR12P) and Chr8p23.1 (HMGB1P46) With Diabetic Neuropathic Pain.” eBioMedicine 2, no. 10: 1386–1393. 26629533 10.1016/j.ebiom.2015.08.001 PMC4634194

[ejp70146-bib-0029] Meng, W. , P. S. Reel , C. Nangia , et al. 2023. “A Meta‐Analysis of the Genome‐Wide Association Studies on Two Genetically Correlated Phenotypes Suggests Four New Risk Loci for Headaches.” Phenomics 3, no. 1: 64–76. 36939796 10.1007/s43657-022-00078-7 PMC9883337

[ejp70146-bib-0030] PAINSTORM . n.d. “The PAINSTORM Project.” https://www.painstorm.co.uk/.

[ejp70146-bib-0031] Pascal, M. M. , A. C. Themistocleous , R. Baron , et al. 2019. “DOLORisk: Study Protocol for a Multi‐Centre Observational Study to Understand the Risk Factors and Determinants of Neuropathic Pain.” Wellcome Open Research 3: 63. 30756091 10.12688/wellcomeopenres.14576.1 PMC6364377

[ejp70146-bib-0032] Porta, M. S. 2014. A Dictionary of Epidemiology. 6th ed. Oxford University Press.

[ejp70146-bib-0033] Rikard, S. M. , A. E. Strahan , K. M. Schmit , and G. P. Guy Jr . 2023. “Chronic Pain Among Adults—United States, 2019–2021.” https://www.cdc.gov/mmwr/volumes/72/wr/mm7215a1.htm. 10.15585/mmwr.mm7215a1 PMC10121254 37053114

[ejp70146-bib-0034] Robinson, P. N. 2012. “Deep Phenotyping for Precision Medicine.” Human Mutation 33, no. 5: 777–780. 10.1002/humu.22080. 22504886

[ejp70146-bib-0035] Rometsch, C. , A. Martin , F. Junne , and F. Cosci . 2025. “Chronic Pain in European Adult Populations: A Systematic Review of Prevalence and Associated Clinical Features.” Pain 166, no. 4: 719–731. 40101218 10.1097/j.pain.0000000000003406 PMC11921450

[ejp70146-bib-0036] Scholz, J. , N. B. Finnerup , N. Attal , et al. 2019. “The IASP Classification of Chronic Pain for ICD‐11: Chronic Neuropathic Pain.” Pain 160, no. 1: 53–59. 30586071 10.1097/j.pain.0000000000001365 PMC6310153

[ejp70146-bib-0037] Smith, B. H. , A. Campbell , P. Linksted , et al. 2013. “Cohort Profile: Generation Scotland: Scottish Family Health Study (GS:SFHS). The Study, Its Participants and Their Potential for Genetic Research on Health and Illness.” International Journal of Epidemiology 42, no. 3: 689–700. 22786799 10.1093/ije/dys084

[ejp70146-bib-0038] Smith, B. H. , A. M. Elliott , W. A. Chambers , W. C. Smith , P. C. Hannaford , and K. Penny . 2001. “The Impact of Chronic Pain in the Community.” Family Practice 18, no. 3: 292–299. 11356737 10.1093/fampra/18.3.292

[ejp70146-bib-0039] Sudlow, C. , J. Gallacher , N. Allen , et al. 2015. “UK Biobank: An Open Access Resource for Identifying the Causes of a Wide Range of Complex Diseases of Middle and Old Age.” PLoS Medicine 12, no. 3: e1001779. 25826379 10.1371/journal.pmed.1001779 PMC4380465

[ejp70146-bib-0040] Swerdel, J. N. , and M. M. Conover . 2023. “Comparing Broad and Narrow Phenotype Algorithms: Differences in Performance Characteristics and Immortal Time Incurred.” Journal of Pharmacy and Pharmaceutical Sciences 26: 12095. 10.3389/jpps.2023.12095. 38235322 PMC10791821

[ejp70146-bib-0041] Tanguay‐Sabourin, C. , M. Fillingim , G. V. Guglietti , et al. 2023. “A Prognostic Risk Score for Development and Spread of Chronic Pain.” Nature Medicine 29, no. 7: 1821–1831. 10.1038/s41591-023-02430-4 PMC10353938 37414898

[ejp70146-bib-0042] Treede, R.‐D. , W. Rief , A. Barke , et al. 2019. “Chronic Pain as a Symptom or a Disease: The IASP Classification of Chronic Pain for the International Classification of Diseases (ICD‐11).” Pain 160, no. 1: 19–27. 30586067 10.1097/j.pain.0000000000001384

[ejp70146-bib-0043] Van Hecke, O. , L. J. Hocking , N. Torrance , et al. 2017. “Chronic Pain, Depression and Cardiovascular Disease Linked Through a Shared Genetic Predisposition: Analysis of a Family‐Based Cohort and Twin Study.” PLoS One 12, no. 2: e0170653. 28225781 10.1371/journal.pone.0170653 PMC5321424

[ejp70146-bib-0044] Van Hecke, O. , P. R. Kamerman , N. Attal , et al. 2015. “Neuropathic Pain Phenotyping by International Consensus (NeuroPPIC) for Genetic Studies: A NeuPSIG Systematic Review, Delphi Survey, and Expert Panel Recommendations.” Pain 156, no. 11: 2337–2353. 26469320 10.1097/j.pain.0000000000000335 PMC4747983

[ejp70146-bib-0045] Veluchamy, A. , H. L. Hebert , W. Meng , C. N. Palmer , and B. H. Smith . 2018. “Systematic Review and Meta‐Analysis of Genetic Risk Factors for Neuropathic Pain.” Pain 159, no. 5: 825–848. 29351172 10.1097/j.pain.0000000000001164

[ejp70146-bib-0046] Veluchamy, A. , H. L. Hébert , N. R. van Zuydam , et al. 2021. “Association of Genetic Variant at Chromosome 12q23.1 With Neuropathic Pain Susceptibility.” JAMA Network Open 4, no. 12: e2136560. 34854908 10.1001/jamanetworkopen.2021.36560 PMC8640893

[ejp70146-bib-0047] Von Korff, M. , J. Ormel , F. J. Keefe , and S. F. Dworkin . 1992. “Grading the Severity of Chronic Pain.” Pain 50, no. 2: 133–149. 1408309 10.1016/0304-3959(92)90154-4

[ejp70146-bib-0048] Zimmer, Z. , K. Fraser , H. Grol‐Prokopczyk , and A. Zajacova . 2022. “A Global Study of Pain Prevalence Across 52 Countries: Examining the Role of Country‐Level Contextual Factors.” Pain 163, no. 9: 1740–1750. 35027516 10.1097/j.pain.0000000000002557 PMC9198107

[ejp70146-bib-0049] Zorina‐Lichtenwalter, K. , C. I. Bango , L. Van Oudenhove , et al. 2023. “Genetic Risk Shared Across 24 Chronic Pain Conditions: Identification and Characterization With Genomic Structural Equation Modeling.” Pain 164, no. 10: 2239–2252. 37219871 10.1097/j.pain.0000000000002922 PMC10524350

